# **α**-Synuclein antisense oligonucleotides as a disease-modifying therapy for Parkinson’s disease

**DOI:** 10.1172/jci.insight.135633

**Published:** 2021-03-08

**Authors:** Tracy A. Cole, Hien Zhao, Timothy J. Collier, Ivette Sandoval, Caryl E. Sortwell, Kathy Steece-Collier, Brian F. Daley, Alix Booms, Jack Lipton, Mackenzie Welch, Melissa Berman, Luke Jandreski, Danielle Graham, Andreas Weihofen, Stephanie Celano, Emily Schulz, Allyson Cole-Strauss, Esteban Luna, Duc Quach, Apoorva Mohan, C. Frank Bennett, Eric E. Swayze, Holly B. Kordasiewicz, Kelvin C. Luk, Katrina L. Paumier

**Affiliations:** 1 Ionis Pharmaceuticals Inc., Carlsbad, California, USA.; 2 Michigan State University, Grand Rapids, Michigan, USA.; 3 Biogen, Cambridge, Massachusetts, USA.; 4 Department of Pathology and Laboratory Medicine, University of Pennsylvania Perelman School of Medicine, Philadelphia, Pennsylvania, USA.

**Keywords:** Neuroscience, Therapeutics, Parkinson disease

## Abstract

Parkinson’s disease (PD) is a prevalent neurodegenerative disease with no approved disease-modifying therapies. Multiplications, mutations, and single nucleotide polymorphisms in the *SNCA* gene, encoding α-synuclein (aSyn) protein, either cause or increase risk for PD. Intracellular accumulations of aSyn are pathological hallmarks of PD. Taken together, reduction of aSyn production may provide a disease-modifying therapy for PD. We show that antisense oligonucleotides (ASOs) reduce production of aSyn in rodent preformed fibril (PFF) models of PD. Reduced aSyn production leads to prevention and removal of established aSyn pathology and prevents dopaminergic cell dysfunction. In addition, we address the translational potential of the approach through characterization of human *SNCA-*targeting ASOs that efficiently suppress the human *SNCA* transcript in vivo. We demonstrate broad activity and distribution of the human *SNCA* ASOs throughout the nonhuman primate brain and a corresponding decrease in aSyn cerebral spinal fluid (CSF) levels. Taken together, these data suggest that, by inhibiting production of aSyn, it may be possible to reverse established pathology; thus, these data support the development of *SNCA* ASOs as a potential disease-modifying therapy for PD and related synucleinopathies.

## Graphical abstract

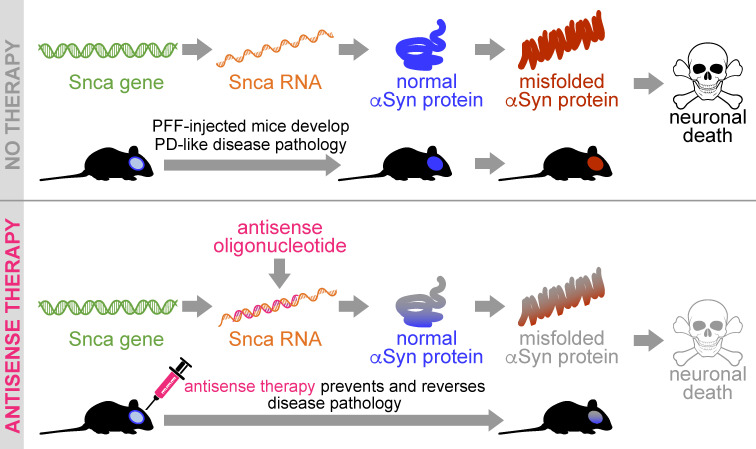

## Introduction

There is strong genetic evidence implicating the role of α-synuclein protein (aSyn) in the pathogenesis of Parkinson’s disease (PD) (1–15). Intracellular aSyn aggregates, pathological hallmarks of PD, increase in number and spread through the brain as symptoms worsen in PD patients (16, 17). Duplication, triplication, or genetic mutations in the *SNCA* gene (which produces aSyn protein; e.g., A53T, A30P, E46K, G51D) are linked to autosomal dominant forms of the disease (1, 3, 4, 10, 13, 18). Moreover, polymorphisms that occur within specific regions of the *SNCA* gene increase the overall risk of PD by either increasing the production or slowing the clearance of aSyn (5, 8, 12, 14, 19, 20). A toxic gain-of-function of aSyn is also established in other synucleinopathies, including multiple-system atrophy (MSA) (21), Diffuse Lewy body disease (DLBD) (22), and Gaucher disease (GD) (23), which collectively affects about 1% of people over 60 years of age. Clinically diagnosed dementia with Lew bodies (DLB) (21) and pure autonomic failure (PAF) (24, 25) also exhibit Lewy pathology, suggesting a toxic gain of function of aSyn in DLB and PAF, making aSyn knockdown a potentially viable therapeutic approach for several patient populations.

To date, there are multiple therapeutic strategies being investigated, including antibodies and small molecule approaches targeting different forms and conformational states of aSyn; however, the toxic species of aSyn has not yet been confirmed, potentially limiting therapeutic benefit of these approaches (26, 27). In addition, antibodies and small molecules often only target extracellular pools of the protein, which could decrease the viability of these approaches. However, antisense oligonucleotide (ASO) therapy can potentially overcome the limitations of these approaches by inhibiting the production of aSyn by targeting *SNCA* RNA intracellularly, thereby reducing all forms of the aSyn protein (28).

To determine the efficacy of ASOs in synucleinopathies, we used rat and mouse aSyn preformed fibril (PFF) transmission models, which replicate aspects of human PD progression, including seeding and aggregate deposition of endogenous aSyn, reduced striatal dopamine, dopaminergic cell dysfunction (tyrosine hydroxylase [TH] loss) in the substantia nigra (SN), and motor dysfunction (29, 30). We hypothesized that these pathological changes could be prevented or reversed by inhibiting the production of aSyn using ASOs targeted to the *Snca* gene. Though aSyn pathology has been shown to be reversible with complete genetic ablation of the *Snca* gene in adult mice in overexpression and toxin models (31–36) and with suppression in cells (37), we aimed to determine if partial transient and/or sustained suppression of endogenous aSyn with a therapeutically relevant approach in vivo could lead to reversal of established pathology and prevention of TH^+^ loss. We also aimed to improve cellular function, of which some aspects have been shown to be dysfunctional in iPSC-derived PD patient neurons and PD patient tissue, as well as in animal models of PD (38–42). For example, the association of DNA damage and neurodegeneration are increasingly recognized as common features of multiple neurodegenerative diseases (43). DNA damage and aSyn pathology are associated with TH^+^ loss and deficits in mitochondrial function (44). Remarkably, we found a robust clearance of aSyn pathology when production of aSyn is inhibited, which results in improvement in cell function as measured by double-stranded DNA breaks, a measure of DNA damage. With human *SNCA* targeting ASOs, we demonstrate widespread target engagement in the brain of transgenic mice expressing human *SNCA* and in all of the PD-relevant brain regions in NHPs, thus demonstrating the therapeutic potential of ASOs for PD and other synucleinopathies.

## Results

### Reduction of Snca improves cellular function in cells and prevents pathogenic aSyn aggregate deposition in an in vivo PFF model of PD.

**Figure 1:**
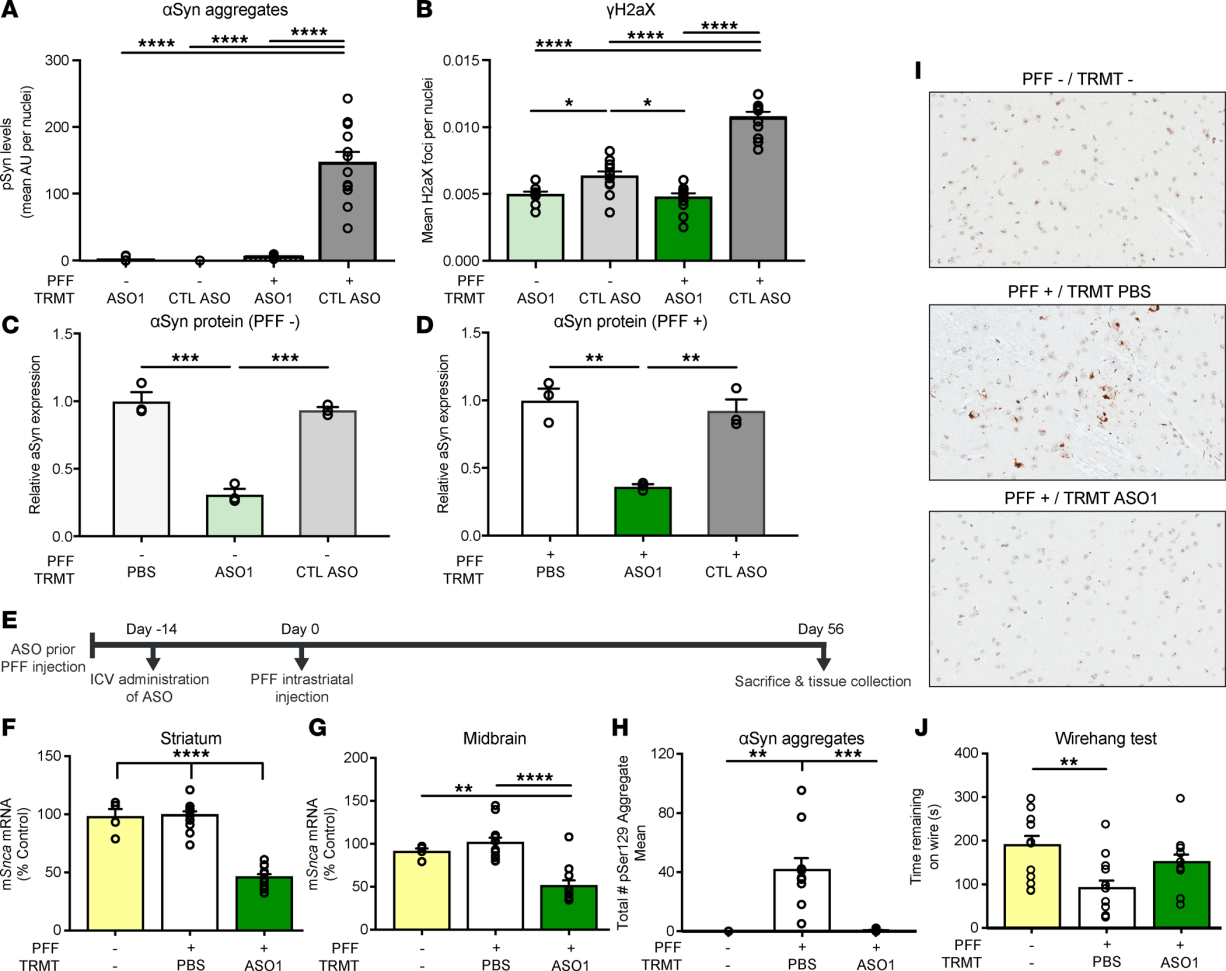
ASO-mediated reduction of *Snca* improves cellular function in cells and prevents pathogenic aSyn aggregate deposition in an in vivo PFF model of PD. (**A** and **B**) Quantification of pSer129^+^ area by intensity in mouse primary cortical cultures and cellular function, by γH2AX Ser139, in mouse primary cortical cultures with either CTL ASO or ASO1 30 minutes following PFF addition. Replicated 2 times. (**C**) Quantification of aSyn protein by Western blot from mouse primary cortical cultures not treated with PFF (*n* = 3). (**D**) Quantification of aSyn protein by Western blot from mouse primary cortical cultures treated with PFF. (**E**) Timeline for single 700 μg i.c.v. bolus ASO administration prior to the PFF injection paradigm with termination at day 56. (**F** and **G**) mRNA reduction by RT-PCR in striatum and midbrain. (**H**) Quantification of pSyn^+^ aggregate reduction (total enumeration) in the substantia nigra by IHC. (**I**) Representative images of immunostaining (IHC) for pSer129+ aggregate counts. Original magnification, 100×. (**J**) Performance on a wire hang task (*n* = 4, 12, and 11 for naive, PBS, and ASO1, respectively, except wire hang, in which *n* = 12 for naive). Data are represented as ± SEM. *P < 0.05, **P < 0.01, ***P < 0.001, ****P < 0.0001 (2-way ANOVA with Tukey post hoc analyses for duration of action with all other analyses using 1-way ANOVA with Tukey post hoc analyses). PFF, preformed fibril; TRMT, treatment.

Two 2’-O-methoxyethyl/DNA gapmer ASOs targeting *Snca* were used for in vitro and in vivo experimentation in addition to a control ASO (Table 1). In previous work, ASO suppression of *Snca* in a primary cortical culture PFF system reduced *Snca* mRNA and reversed phospho-Ser129 (pSer129^+^) pathology and cellular dysfunction (37). To examine an additional capability of ASOs to improve cellular function, we utilized this same ASO (ASO1) and system. As previously published, double-stranded DNA breaks were increased with PFFs, corresponding with aSyn pathology, TH^+^ loss, and mitochondrial dysfunction (44). Thus, we sought to characterize double-stranded DNA breaks to determine whether an *Snca* ASO could mitigate dysfunction. As expected, application of PFFs resulted in production of pSer129^+^ aggregates in primary mouse cortical cultures causing double-stranded breaks (γH2AX Ser139) (Figure 1, A and B). Remarkably, administration of *Snca* ASO1 was able to reduce pSer129^+^ pathology, lower aSyn protein, and mitigate double-stranded breaks (γH2AX Ser139) but not a control ASO (Figure 1, A–D).

To further extend this work, we assessed *Snca* ASOs in a rodent model of PD. Rodent aSyn PFF intrastriatal injection models result in accumulation of pS129^+^ aggregates that propagate to interconnected regions (including from the SN to the striatum), leading to dysfunction of the nigrostriatal system (29, 30). A single 700 μg intracerebroventricular (i.c.v.) injection of *Snca*-targeted ASO1 reduced *Snca* mRNA by approximately 50% 70 days after ASO administration (Figure 1, E–G); this resulted in prevention of pSer129^+^ aggregate deposition in the PFF mouse model (approximately 96% reduction; Figure 1, H and I) 56 days following PFF injection. PFF-injected mice exhibit significant deficits in a wire hang motor function task, as published previously (29). Following administration of ASO1, mice that were injected with PFFs no longer exhibit a significant deficit in comparison with naive mice (Figure 1J).

### ASO-mediated reduction of Snca is dose responsive, exhibits a prolonged duration of action, and prevents pathogenic aSyn aggregate deposition in an in vivo PFF model of PD.

**Figure 2:**
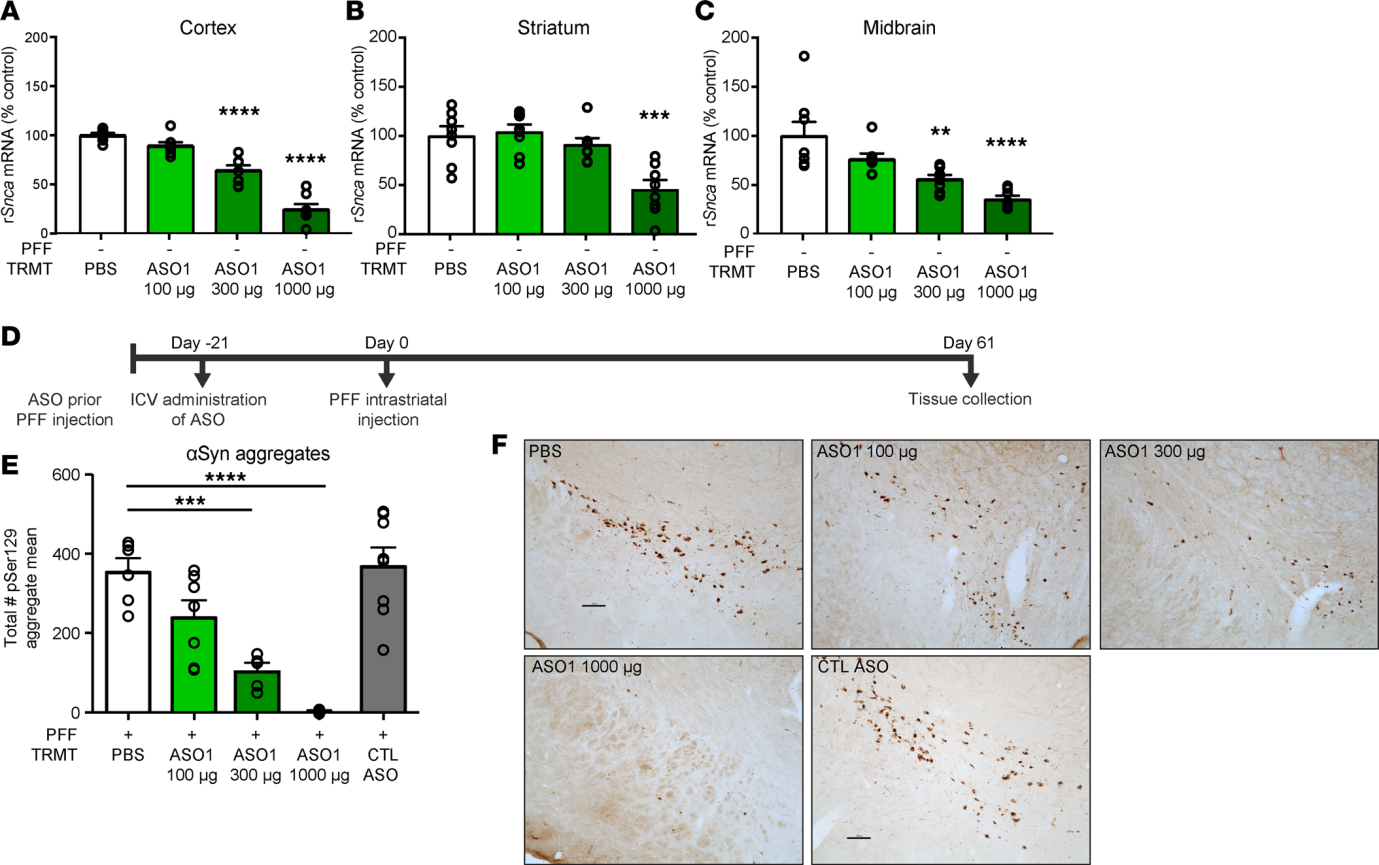
ASO-mediated reduction of *Snca* is dose responsive and prevents pathogenic aSyn aggregate deposition in an in vivo PFF model of PD. (**A**–**C**) Three-week dose response of rat *Snca* levels from cortical, striatal, and midbrain rat samples by qPCR (*n* = 8 per dose). (**D**) Timeline for ASO dose-response administration prior to PFF injection paradigm. (**E**) Quantification of immunostaining for pSer129^+^ aggregate counts by total enumeration (*n* = 6, 7, 5, 8, 8 for PBS, 100 μg, 300 μg, 1000 μg, and CTL ASO, respectively). (**F**) Representative images of pSer129^+^ aggregate counts in the substantia nigra. Scale bar: 100 μm. Data are represented as ± SEM. **P < 0.01, ***P < 0.001, ****P < 0.0001 (2-way ANOVA with Tukey post hoc analyses for duration of action, with all other analyses using 1-way ANOVA with Tukey post hoc analyses). PFF, preformed fibril; TRMT, treatment; CTL ASO, control ASO.

More extensive characterization of ASO1 demonstrated dose-dependent reduction of *Snca* mRNA in vivo 21 days following a single i.c.v. administration in rats (Figure 2, A–C). Dose-dependent ASO-mediated *Snca* mRNA suppression with ASO1 also resulted in dose-dependent prevention of pSer129^+^ aggregate deposition in comparison with the PBS group at 61 days after PFF injection (82 days after ASO i.c.v. administration) in the SN (Figure 2, D–F, and Supplemental Figure 1, A and B; supplemental material available online with this article; https://doi.org/10.1172/jci.insight.135633DS1). This is consistent with complete prevention of aggregates in aSyn-KO mice and attenuated aggregation in heterozygous KO mice injected with PFFs (29). There was no evidence of dopaminergic cell dysfunction at this time point (Supplemental Figure 1C), as expected (30), and the control ASO exhibited a profile similar to PBS administered rats, indicating that the control ASO does not alter disease (Figure 2, E and F, and Supplemental Figure 1, A–C). ASO1 reduced *Snca* mRNA and prevented pSer129^+^ aggregate deposition in the SN and across multiple brain regions, including prefrontal cortex and motor cortex 61 days after PFF injection (Supplemental Figure 1, D–G).

A second *Snca*-targeting ASO (ASO2) was also evaluated. *Snca* ASO2 also reduced pSer129^+^ aggregate counts but to a lesser extent than ASO1 (Supplemental Figure 1, F and G). This is consistent with ASO2 being a less potent molecule than ASO1, with almost no *Snca* mRNA suppression remaining 82 days after ASO administration (Supplemental Figure 1, D and E) and more modest *Snca* mRNA suppression than ASO1 at 42 days after ASO administration (Supplemental Figure 1, H–J). Taken together, these data suggest that aSyn pathology is dependent on aSyn expression levels.

### ASO-mediated suppression of Snca prevents dopaminergic cell dysfunction in an in vivo PFF model of PD.

**Figure 3:**
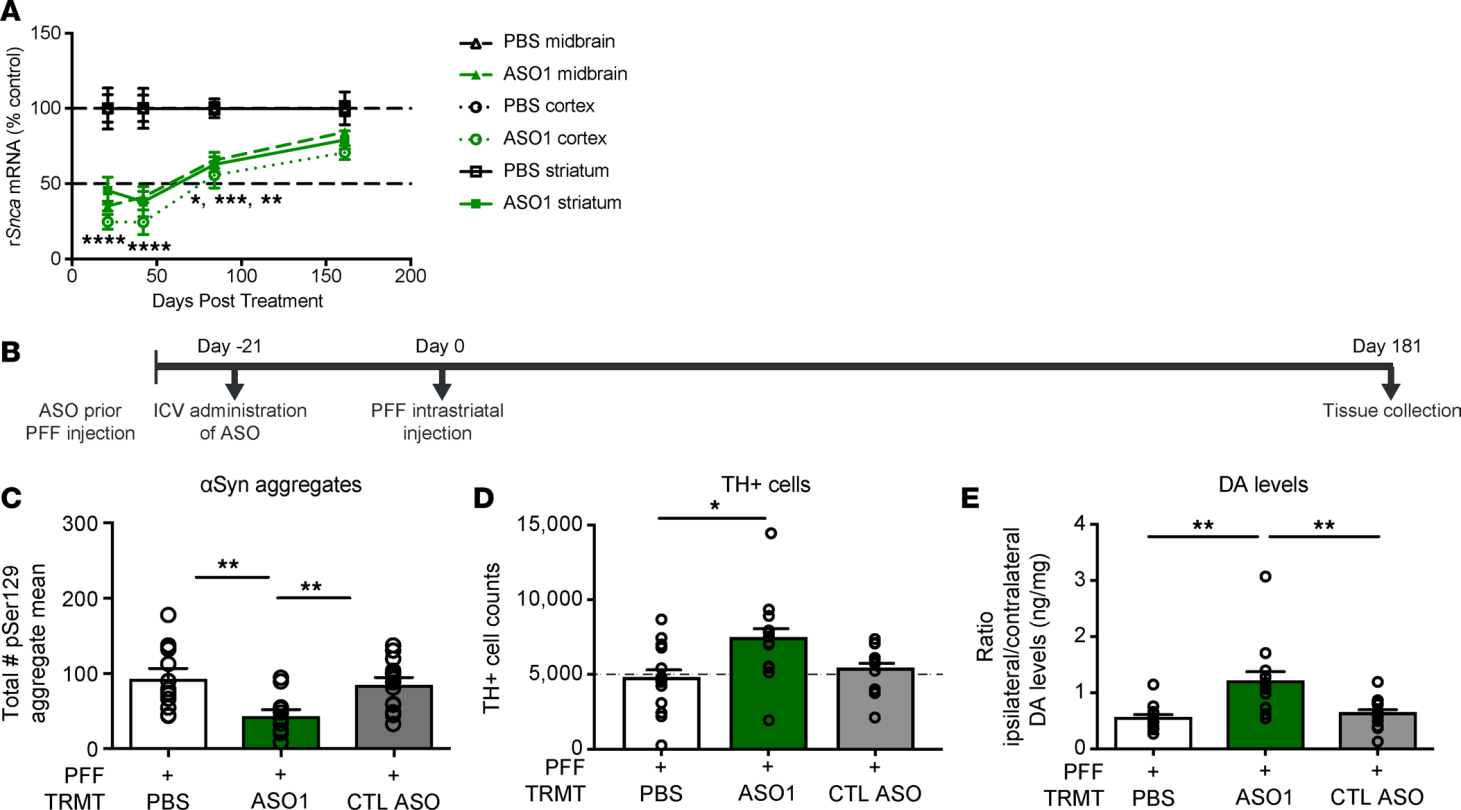
ASO-mediated suppression of *Snca* exhibits a prolonged duration of action and prevents dopaminergic cell dysfunction in an in vivo PFF model of PD. (**A**) Time course of *Snca* mRNA reduction (the 1000 μg results for the 3-week time point in Figure 2, A–C are included). (**B**–**E**) Results from ASO administration (1000 μg) prior to PFF injection paradigm in rats with study termination at 181 days. (**B**) Timeline for ASO administration prior to PFF injection paradigm in rats. (**C**–**E**) pSer129^+^ aggregate counts using total enumeration by IHC (*n* = 13, 13, and 15 for PBS, ASO1, and CTL ASO, respectively), dopaminergic cell counts by IHC (by stereology) (*n* = 13, 13, and 12 for PBS, ASO1, and CTL ASO, respectively), and striatal dopamine levels by HPLC normalized to the contralateral side (*n* = 13, 13, and 14 for PBS, ASO1, and CTL ASO, respectively). Data are represented as ± SEM. *P < 0.05, **P < 0.001, ***P < 0.0001, ****P < 0.00001 (1-way ANOVA with Tukey post hoc analyses). PFF, preformed fibril; TRMT, treatment; CTL ASO, control ASO. Statistical significance was also achieved when using nonparametric test Kruskall-Wallis for Figure 3, D and E; *P* ≥ 0.02. The same animal was high for TH and DA levels.

PFF rodent models, like the human condition, undergo neurodegeneration in a number of CNS regions after pathology is established (16, 29, 30); this typically takes about 4–6 months after PFF injection. Fortunately, ASO1 exhibits a long duration of action with significant target suppression up to 84 days in the cortex, striatum, and midbrain, returning to baseline approximately 160 days after a single i.c.v. administration at 1000 μg (Figure 3A). To determine if ASO-mediated suppression of aSyn production could prevent TH loss, rats were administered a single i.c.v. 1000 μg dose of ASO1 21 days prior to PFF injection and assessed 202 days following i.c.v. ASO administration (Figure 3B). ASO1 administration resulted in significant reduction of pSer129^+^ aggregates in comparison with PBS (approximately 53% reduction) and control ASO–treated (approximately 48% reduction) rats (Figure 3C); it also resulted in significantly attenuated PFF-mediated TH loss in the SN compared with PBS at 202 days after a single ASO administration (Figure 3D; contralateral TH^+^ quantification in Supplemental Figure 2C). Striatal dopamine levels were also normalized, in comparison with the respective contralateral side, with ASO1 treatment compared with PBS or control ASO (Figure 3E). *Snca* mRNA had returned to normal levels at this 202 day time point, 181 days after PFF injection (in comparison with PBS treated rats), which likely explains the modest accumulation of aSyn aggregates when compared with almost complete ablation of pathology at the 61 day time point when aSyn production was still suppressed (Supplemental Figure 2, A and B, in comparison with Supplemental Figure 1, A, B, D, and E; TH^+^ cells were not yet reduced at this time point; Supplementary Figure 1C).

### Pathogenic aSyn aggregate deposition is reversible, and its amelioration prevents TH loss.

**Figure 4:**
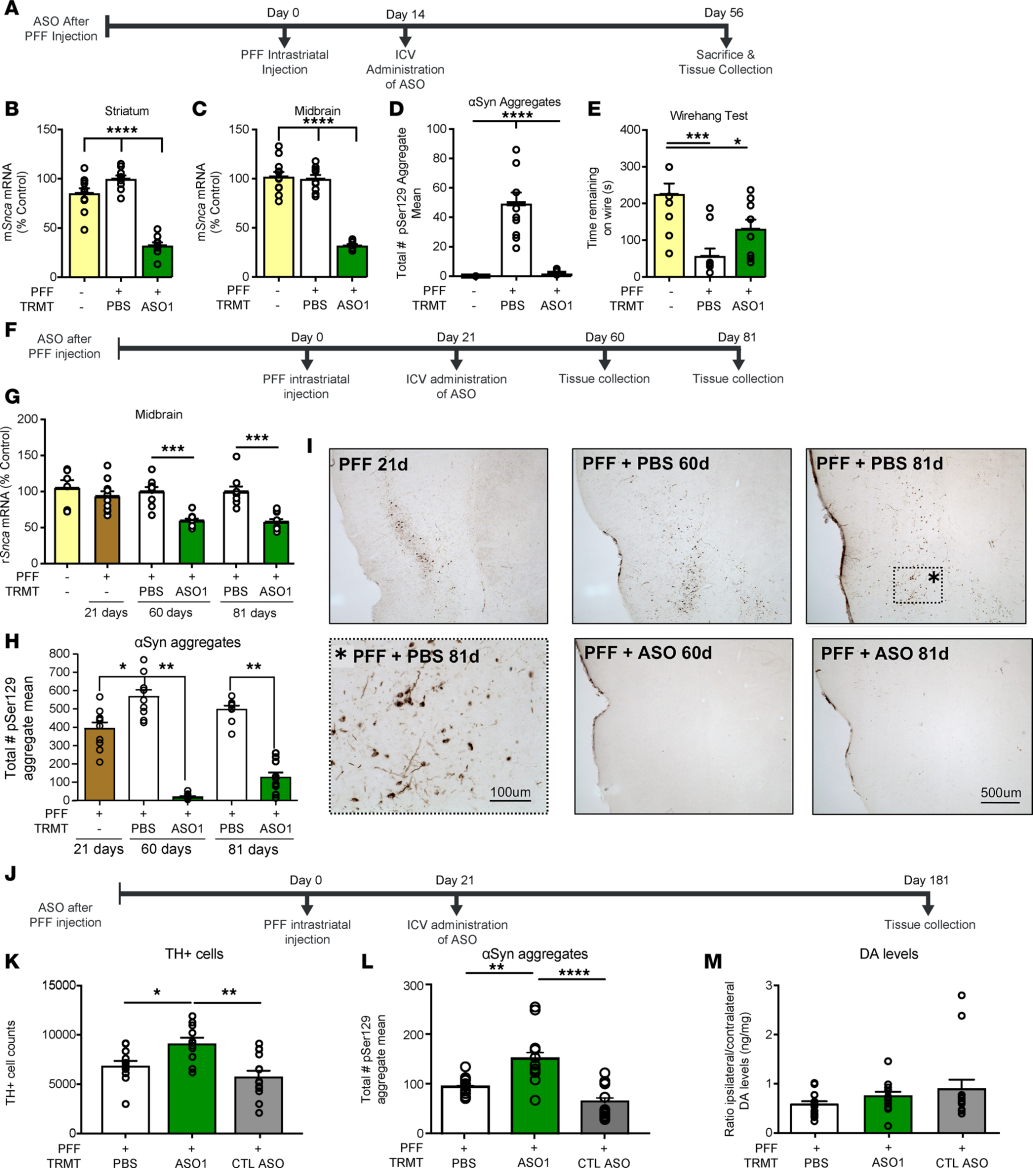
Pathogenic aSyn aggregate deposition is reversible, and its amelioration prevents TH loss. (**A**) Timeline for ASO administration after PFF injection paradigm in the mouse. (**B** and **C**) mRNA reduction by RT-PCR (*n* = 12, 10, and 10 for naive, PBS, and ASO1, respectively) (**D** and **E**) Quantification of aggregate reduction in the substantia nigra by IHC (*n* = 4, 10, and 10 for naive, PBS, and ASO1, respectively) and performance on a wire hang task (*n* = 10, 10, and 10 for naive, PBS, ASO1, respectively). (**F**) Timeline for ASO administration (1000 μg) for **G**–**I**. (**G**) Quantification of *Snca* mRNA reduction in the midbrain at each time point. (**H** and **I**) Quantification of immunostaining for pSer129^+^ aggregate counts in the midbrain, and representative images from the insular cortex (*n* = 10, 9, 9, 9, and 10 for PFF only, PBS 60 days, ASO1 60 days, PBS 81 days, and ASO1 81 days, respectively). (**J**–**M**) Results from ASO administration (1000 μg) with study termination at 181 days. (**J**) Timeline for ASO administration for **K**–**M**. (**K**) TH^+^ cell counts by IHC (by stereology) (*n* = 12, 11, and 14 for PBS, ASO1, and CTL ASO, respectively). (**L** and **M**) Quantification of pSer129^+^ aggregate counts in the substantia nigra by IHC (*n* = 13, 12, and 12 for PBS, ASO1, and CTL ASO, respectively) and striatal dopamine levels by HPLC normalized to the contralateral side (*n* = 13, 12, and 14 for PBS, ASO1, and CTL ASO, respectively). Data are represented as ± SEM. *P < 0.05, **P < 0.001, ***P < 0.0001, ****P < 0.00001 (1-way ANOVA with Tukey post hoc analyses). PFF, preformed fibril; TRMT, treatment; CTL ASO, control ASO.

To determine if ASO-mediated *Snca* suppression could be beneficial after established pathology, ASO1 was administered by i.c.v. bolus at 700 μg 14 days after PFF injection in mice, a time point with established pSer129^+^ aggregates in the SN (Figure 4A). *Snca* mRNA was significantly reduced in the striatum and midbrain 56 days after PFF injection (Figure 4, B and C). Strikingly, ASO1 reduced the number of established aggregates (approximately 92% reduction; Figure 4D) 56 days after PFF injection. A trend toward improvement in a wire hang behavioral task was also found in mice (Figure 4E). In addition, ASO1 was administered by i.c.v. bolus at 1000 μg 21 days after PFF injection in rats, a time point with established pSer129^+^ aggregates in the SN (Figure 4, F and H). *Snca* mRNA reduction was as expected following ASO treatment (Figure 4G). Though maximal PFF deposition occurs at 60 days after injection, rats euthanized 21 days after PFF injection exhibited extensive aggregate deposition that was resolved by ASO administration approximately 40 days later (Figure 4H). Strikingly, ASO1 reduced the number of established aggregates at 60 (approximately 90% reduction) and 81 (approximately 51% reduction) days after PFF injection (39 and 61 days after ASO administration) compared with pSer129^+^ aggregate counts at 21 days (PFF only) in the midbrain. Similar reductions in pSer129^+^ aggregates were found in the insular cortex, confirming widespread activity of the ASO (Figure 4I). Thus, in both mice and rats, ASO1 resulted in reversal of deposition of established aggregates.

To determine if suppression of aSyn after established aggregate deposition could prevent TH loss, rats received a single 1000 μg i.c.v. injection of ASO1 administered 21 days after PFF injection and assessed at 181 days after PFF (160 days after ASO administration) (Figure 4J). ASO1 significantly attenuated PFF-mediated TH^+^ cells in ASO1-treated animals (Figure 4K; contralateral TH^+^ cells in Supplemental 2F). Interestingly, ASO1 administration resulted in a significant increase (approximately 22%) in pSer129^+^ aggregates compared with PBS- and control ASO–administered rats at 181 days after PFF injection (Figure 4L), possibly due to reduced dysfunction of aggregate-bearing neurons. In this cohort, there were no significant differences in striatal dopamine levels for any of the treatment groups (Figure 4M). The increase in aggregates in ASO1-administered animals relative to PBS 160 days after a single ASO administration is consistent with aSyn levels returning to normal over time (Supplemental Figure 2, D and E) and likely higher than PBS due to the preservation of TH cells in ASO1-treated animals. Remarkably, the transient reduction in aSyn production after aggregates were established was sufficient to preserve TH expression in dopaminergic neurons, highlighting the therapeutic value of directly targeting the underlying disease.

### Sustained reduction of Snca with ASO administered after aggregates are established reduces aggregate pathology and prevents TH loss.

**Figure 5:**
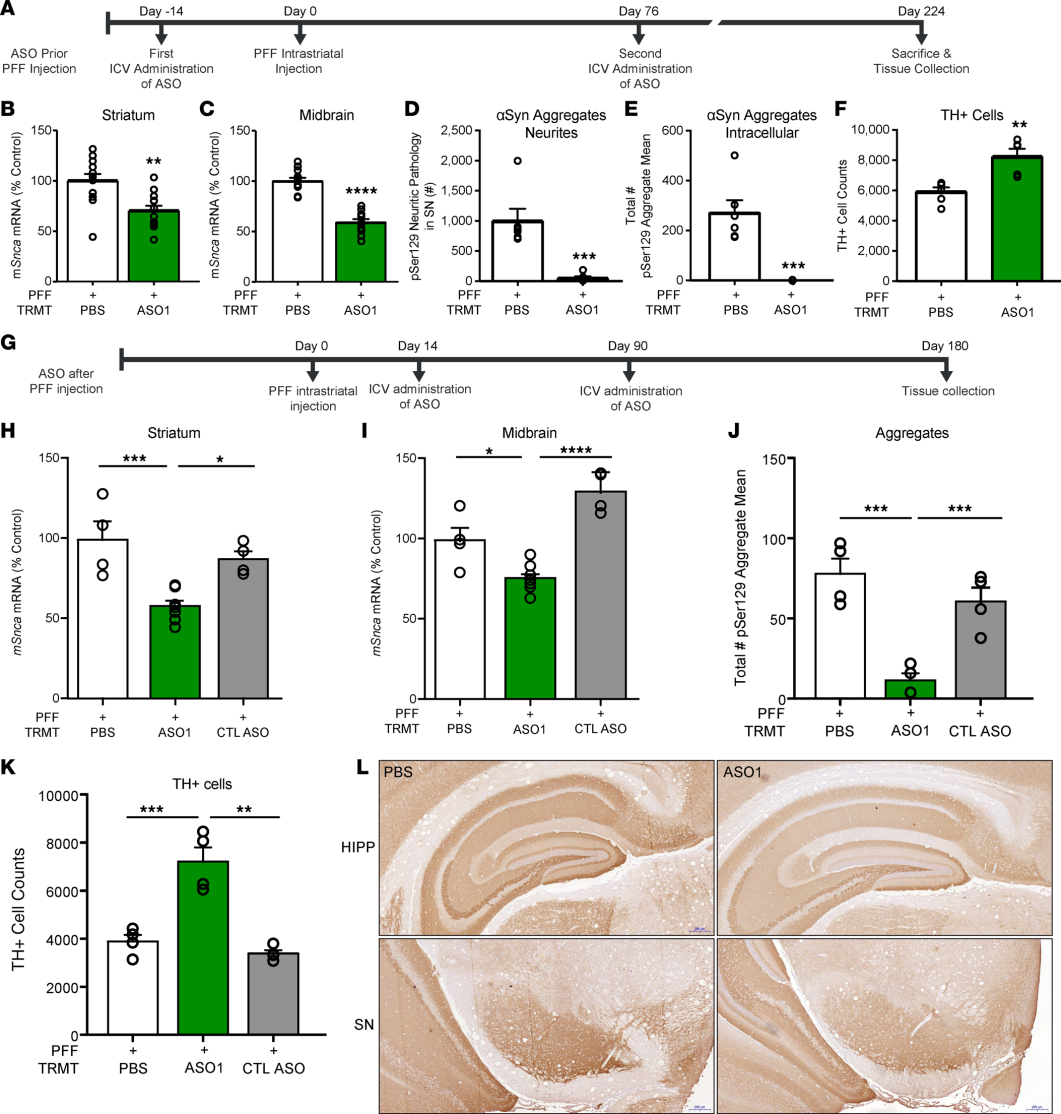
Sustained reduction of *Snca* with ASO administered prior to and after establishment of aggregates reduces aggregate pathology and prevents TH loss. (**A**–**F**) Results from ASO administration prior to PFF injection paradigm with sustained suppression and study termination at 224 days after PFF injection in mice. (**A**) Timeline for ASO administration (700 μg) prior to PFF injection paradigm. (**B** and **C**) mRNA reduction in the striatum and midbrain. (**D** and **E**) pSer129^+^ aggregate counts quantified by IHC in neurites and cell bodies. (**F**) Dopaminergic cell counts quantified by IHC. (*n* = 12 for PBS and ASO1; *n* = 6 for IHC endpoints). (**G**–**K**) Results from ASO administration after PFF injection paradigm with sustained suppression and study termination at 224 days after PFF injection in mice (700 μg). (**G**) Timeline for ASO administration (700 μg) after PFF injection paradigm. (**H** and **I**) mRNA reduction in the striatum and midbrain (*n* = 4, 6, 4, respectively, for PBS, ASO1, and CTL ASO). (**J**) pSer129^+^ aggregate counts quantified by IHC (*n* = 4). (**K**) Dopaminergic cell counts quantified by IHC. (**L**) Representative images of aSyn IHC from coronal sections (*n* = 4). Scale bar: 20 µm. Data are represented as ± SEM. *P < 0.05, **P < 0.001, ***P < 0.0001, ****P < 0.00001 (1-way ANOVA with Tukey post hoc analyses). PFF, preformed fibril; TRMT, treatment; CTL ASO, Control ASO.

To evaluate a paradigm of sustained *Snca* reduction, mice were administered 2 i.c.v. bolus 700 μg administrations of ASO1 at 14 days prior to and 76 days after PFF injection, which resulted in a sustained reduction in *Snca* mRNA, with an approximately 50% reduction remaining 224 days after PFF injection and 238 days after the initial ASO administration (Figure 5, A–C). Sustained *Snca* mRNA reduction resulted in sustained reduction in aSyn pathology and a prevention of TH loss 224 days after PFF injection (Figure 5, D–F). *Snca* mRNA and pSer129^+^ aggregate reduction were similar to the approximately 60-day mouse (Figure 1, E–I) and rat studies (Figure 2 and Supplemental Figure 1, A–J), though tissues were collected 224 days after PFF injection. Thus, maintaining *Snca* suppression prevented aggregates from accumulating and prevented TH loss.

To determine if prolonged ASO-mediated *Snca* suppression could be more beneficial than transient reduction after established pathology, ASO1 was administered i.c.v. at 14 and 90 days following PFF injection, with study termination at 180 days after PFF in mice (Figure 5G). *Snca* mRNA and pSer129^+^ aggregates were reduced in the SN (Figure 5, H–J). TH^+^ loss was also prevented with prolonged aSyn reduction after PFF administration, in comparison with PBS-administered mice (Figure 5K). aSyn pSer129^+^ aggregate reduction and mRNA reduction corresponded to a reduction in aSyn protein, as evaluated histologically with an anti-aSyn antibody (Figure 5L). This differs from single i.c.v.–dose injection following PFF in which aggregate number was increased in ASO1-treated rats in comparison with PBS-treated rats 180 days following PFF injection when mRNA expression had returned to baseline levels (Figure 4L). Thus, therapeutic treatment with *Snca* ASOs administered with established aggregates requires sustained reduction of *Snca* to prevent aggregates from accumulating once *Snca* RNA levels return to baseline.

### Human SNCA ASOs are potent and suppress aSyn broadly in the primate CNS, and CSF aSyn is a potential pharmacodynamic biomarker.

**Figure 6:**
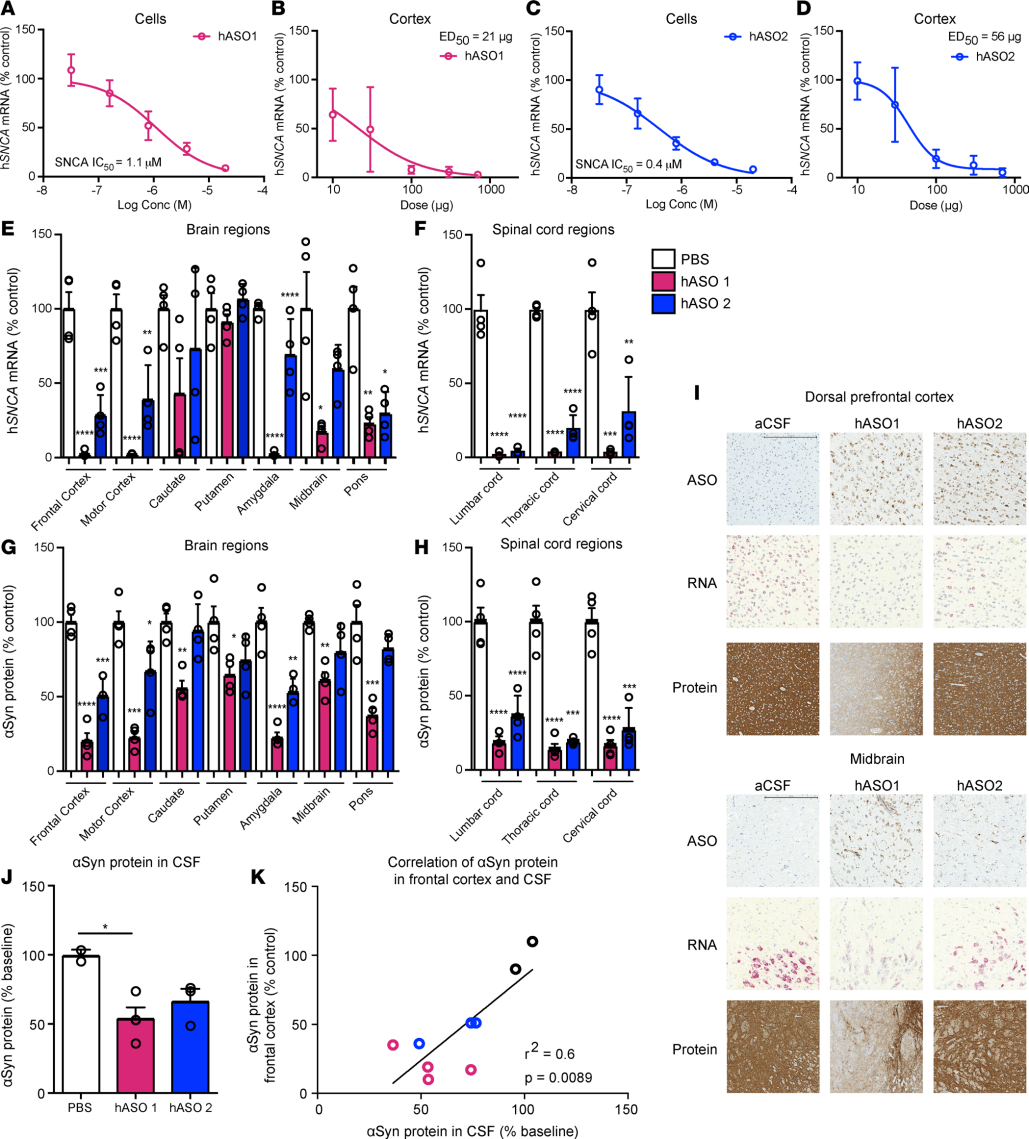
Human *SNCA* ASOs are potent, suppress aSyn broadly in the primate CNS, and CSF aSyn is a suitable pharmacodynamic biomarker. (**A**–**D**) In vitro dose response of human *SNCA* hASO1 and hASO2 in SHSY5Y cells and in vivo dose response in SNCA-PAC mouse cortex 2 weeks after injection (*n* = 10, except 700 μg *n* = 7 for hASO1; *n* = 8 for hASO2 [2 mice removed with ROUT analysis from 300 μg and 700 μg groups], combination of 2 studies of *n* = 4 each; *n* = 12 for all PBS groups). (**E**–**H**) mRNA by RT-PCR and protein by ELISA throughout the brain and spinal cord of the NHP (*n* = 4 for PBS, hASO1, and hASO2). (**I**) Representative images for IHC (ASO and aSyn antibody) or ISH (*SNCA* mRNA). Scale bar: 600 μm for protein/300 μm for ASO and ISH results. (**J** and **K**) Quantification of aSyn protein by ELISA in CSF with correlation of aSyn protein levels in the frontal cortex and CSF (*n* = 2, 3, and 4 for PBS, hASO1, and hASO2, respectively). Data are represented as ± SEM, except for SHSY5Y, which are presented as mean ± SD. *P < 0.05, **P < 0.001, ***P < 0.0001, ****P < 0.00001 (1-way ANOVA with Tukey post hoc analyses).

The ASOs used thus far target the rodent *Snca* transcripts. To treat human patients, we designed ASOs to suppress the human *SNCA* transcript, using similar designs and chemistries as ASOs already in clinical testing (45, 46). Human sequence–targeting *SNCA* ASOs hASO1 and hASO2 (Table 2) suppressed human *SNCA* mRNA in a dose- and concentration-dependent manner (Figure 6, A–D, and Supplemental Figure 3, A and B) in vitro in SH-SY5Y human cells and in vivo in transgenic mice expressing full-length human aSyn (similar to ref. 47). The human *SNCA* ASOs also exhibited an extended duration of action lasting 10 weeks after a single administration (Supplemental Figure 3, C–E). To determine the activity and distribution of human ASOs in a larger brain, hASO1 and hASO2 were given i.t. in nonhuman primates (NHPs, cynomolgus). Following repeated i.t. delivery of hASO1 or hASO2 to the NHPs, *SNCA* mRNA (quantitative PCR; qPCR) and aSyn protein (ELISA) were reduced throughout the brain and spinal cord (Figure 6, E–H). Further analyses of NHP brain tissue by in situ hybridization (*SNCA* mRNA) and IHC (ASO and aSyn protein) support the conclusion that *SNCA* ASOs distribute broadly and result in reduction of *SNCA* mRNA and protein throughout the brain and spinal cord (Figure 6I and Supplemental Figure 4), including regions implicated in PD. aSyn protein in the CSF is significantly reduced with hASO1 in comparison with vehicle-administered (aCSF-administered) NHPs (Figure 6J). In addition, aSyn protein reduction in the frontal cortex correlates with aSyn protein reduction in the CSF, suggesting the use of aSyn in the CSF as a potential pharmacodynamic biomarker (Figure 6K).

## Discussion

Our study demonstrates that ASO-mediated suppression of *Snca* prevented and reversed the progression of aSyn-mediated pathology in rodent transmission models of PD, demonstrating the potential of *SNCA* ASOs as a therapy for PD patients. Long-term studies with sustained reduction of aSyn prevented and even delayed pathology and associated TH loss. Furthermore, central delivery of human *SNCA* ASOs reduced expression of mRNA and protein throughout the brains of both the humanized mouse and NHPs, demonstrating that human *SNCA* ASOs are active in regions of the brain susceptible to PD in a larger species. In NHPs, the reduction of aSyn was reflected in the CSF, supporting further investigation of the use of aSyn protein levels in the CSF as a target engagement biomarker to allow evaluation of efficacy of human *SNCA* ASOs in the clinic.

PD and other synucleinopathies are generally characterized as toxic gain of function in *SNCA*, suggesting that therapeutics designed to lower aSyn production would be beneficial to patients (7, 48–50). To date, there are multiple protein-based therapeutic strategies being investigated targeting different forms and conformational states of aSyn in clinical trials (27). However, the toxic species of aSyn has not yet been confirmed, potentially limiting the therapeutic benefit of these approaches (26, 27). In addition, protein-targeting therapies hypothesized to clear aSyn aggregates as they are transmitted from cell to cell may not be as effective in targeting intracellular Lewy body inclusions. An ASO therapy can potentially overcome these limitations by targeting mRNA intracellularly and reducing all forms of aSyn protein (28, 51). Indeed, ASO-mediated suppression of aSyn reduced aSyn pathology in a dose-dependent manner. In all cases, the levels of aSyn pathology corresponded to the levels of endogenous aSyn production. An ASO less effective at suppressing *Snca* mRNA (ASO2) was less effective at suppressing aSyn pathology. If the *Snca* mRNA levels were allowed to return to normal levels, the aSyn pathology returned; however, this increase was prevented with continued suppression of aSyn production. Taken together, these data illustrate the link between production of endogenous aSyn and aSyn pathology.

Our observation that ASO treatment was beneficial in reducing the number of aSyn aggregates at a time when preexisting pathology is present is particularly interesting. This is consistent with previous work reporting reversal of deposition of soluble aSyn, as well as insoluble forms of pathology following reduction of aSyn production by tetracycline-controlled transactivator (tTA) to prevent expression of the A53T mutant aSyn transgene (32). In the genetic study, stopping production also reversed detrimental changes in hippocampal synaptic markers and hippocampal memory deficits (32). Our results replicate and extend these findings with a therapeutically relevant modality and in a model where we are targeting endogenous aSyn, rather than a transgene. In both cases, stopping aSyn production had a dramatic effect on aSyn pathology and neuronal health. This finding may suggest that de novo production and recruitment of aSyn is required for maintaining the stability of aggregates until a threshold is reached for irreversible cell death. This concept remains to be explored. Regardless, these data suggest that treatment initiation after disease onset in sporadic patients has the potential to reverse disease.

Many toxin, genetic, viral-mediated, and aSyn-injection PD animal models have been generated to evaluate the role of aSyn in PD (52–54). Each model exhibits advantages and limitations (53). The aSyn PFF injection models, used here, exhibit advantages in modeling sporadic PD by relying on fibril seeding and templating of normal endogenous levels of aSyn in specific interconnected brain circuits, avoiding the global overexpression produced in germline transgenic models and the forced local supraphysiological overexpression common in viral vector models (55). Evidence continues to accrue reinforcing prion-like propagation of aSyn aggregates in synucleinopathies, which is reproducible in the PFF injection models, thus, supporting use of this animal model (56). In addition, the progression of aggregate formation in morphology (diffuse to punctate to compact) and important characteristics of Lewy bodies (e.g., proteinase-K resistance, Thioflavin-S^+^) progress over time in the PFF model to result in DA neuron degeneration at 6 months after PFF injection and provide a platform for analyzing the potential of neuroprotective therapies, such as ASOs, for translation to treatment of idiopathic PD. In this regard, our findings are promising.

Few tolerability concerns exist for lowering *SNCA*, as evidenced by genetically engineered *Snca*-deficient mice (57–61). There are reports of cell death in vivo using an shRNA against *Snca* (62, 63), which was not found here with ASOs or in other publications using siRNAs, shRNAs, or ASOs against *Snca* (35, 36, 64). The aSyn antibodies, Prasinezumab (PRX002) and Cinpanemab (BIIB054), completed first-in-human trials in which no serious adverse events were found with aSyn protein lowering (65, 66). In addition, our animal model studies and others (34, 36, 67, 68) have shown a benefit with less than 50% reduction of *Snca* mRNA, indicating that only a partial reduction of *SNCA* will be needed for therapeutic benefit, as mentioned above.

aSyn protein exhibits a profile desirable for a pharmacodynamic biomarker, since reduction in aSyn protein in the CSF correlated with aSyn protein reduction in the frontal cortex in the NHP. aSyn protein is measurable in human CSF and has been shown in patients to not correlate with clinical progression, and its levels are relatively stable over 24 months, suggesting aSyn levels in the CSF may potentially be used in clinical trials as a pharmacodynamic marker to support demonstration of efficacy of aSyn modulating therapies (69–73). Continued evaluation and improved methods for detecting aSyn in the CSF and other fluids are warranted, such as electrochemiluminescense-based detection (74).

ASOs represent a therapeutic approach for directly lowering *SNCA* production because ASOs are sequence specific and can reach CNS targets by i.t. delivery. The ASO platform has become a therapeutic strategy for the treatment of CNS diseases due partly to advances in ASO design, which have improved stability, affinity, potency, and tolerability (28, 51). ASOs have also been shown to distribute widely within the brain and spinal cord in the NHP shown here and elsewhere (75–77), in addition to exhibiting a long duration of effect (78–82). Feasibility of the approach is supported by FDA approval of Spinraza for the treatment of spinal muscular atrophy (83, 84), the recently completed clinical trial with an ASO for Huntington’s (Htt) disease (46) and ongoing trials for an SNCA-targeted ASO for synucleinopathy (NCT04165486), a SOD1-targeted ASO for amyotrophic lateral sclerosis (NCT02623699), a C9ORF72-targeted ASO for ALS (NCT03626012), a LRRK2-targeted ASO for PD (NCT03976349), and a MAPT-targeted ASO therapy for Alzheimer’s disease (NCT03186989). To explore the translational potential of an *SNCA* ASO therapy, we examined target engagement of human *SNCA* targeting ASOs in a human cell line, a human aSyn transgenic mouse model, and NHPs. The *SNCA* targeting molecules described here have sufficient potency and duration of action for use in human patients. Thus, ASOs designed against human *SNCA* have the potential to be a disease-modifying therapeutic for PD patients.

## Methods

### Oligonucleotide synthesis.

The synthesis and purification of all lyophilized ASOs was formulated in PBS without Ca/Mg (Thermo Fisher Scientific, 14190) as previously described and stored at –20°C (85, 86). Sequences and chemistries used are listed in Tables 1 and 2, where capital letters indicate base abbreviation, m indicates 5-methylcytosine, e indicates 2′-O-methoxyethylribose (MOE), k indicates (S)-2′,4′-constrained 2′-O-ethyl (cEt), d indicates deoxyribose, s indicates phosphorothioate, and o indicates phosphodiester.

### Cell culture PFF experiments.

Timed-pregnant CD1 mice (Charles River Laboratories) were utilized for primary neuronal cultures. Hippocampal neurons were prepared from embryos (E16–E18) as previously described (87). All other methods, including PFF generation, PFF treatment, and ASO addition to cultures, were performed as previously described (37).

PSer129^+^ pathology and double-stranded DNA breaks by γH2AX Ser139 were quantified as previously described (42).

### Rats.

Adult male Sprague Dawley rats (200–225 g; Harlan Laboratory) were utilized in rat experiments. Rat PFF studies were conducted at Michigan State University.

### Intrastriatal PFF injections in rats.

Purification of recombinant in vitro fibril assembly was performed as previously described (30). Animals were monitored weekly following surgery and sacrificed at various time points.

### I.c.v. injection in rats.

Rats were injected into the right cerebral ventricle (i.c.v.) using a stereotactic device. For i.c.v. bolus injections, the coordinates were –1.0 mm anterior/posterior and 1.5 mm to the right medial/lateral. The needle was lowered –3.7 mm dorsal/ventral. The proper amount of injection solution (30 μL) was injected by hand at injection rates of approximately 1 μL/second with a 5-minute wait following completion of injection. The incision was sutured closed using 1 horizontal mattress stitch with 3-*O* Ethilon suture. The animals were then allowed to recover from the anesthesia in their home cage.

### Tissue processing in rats.

Following in-life completion, rats were euthanized by CO_2_ asphyxiation. Sections of spinal cord (2 mm) and different brain regions were collected for mRNA analysis. Brains perfused with physiological saline were cooled in iced saline and cut coronally in a 2 mm–thick slab immediately rostral to the hypothalamus. The section was placed onto a petri dish on ice and further dissected into 3 pieces each for striatum and overlying cortex. Dorsal lateral striatum was collected for HPLC, dorsal intermediate striatum was collected for RNA, and dorsal medial striatum was collected for protein. Similarly, medial cortex was collected for HPLC, intermediate cortex was collected for mRNA, and lateral cortex was collected for protein. Dissections were optimized for HPLC analysis corresponding to the pattern of dopamine innervation. Each dissected region was frozen at –80°C until analysis. IHC, immunofluorescence, pSer129^+^ aggregate counts (total enumeration), and stereology were performed as previously described (30).

### IHC in rats.

Primary mouse anti-pSyn (81a, monoclonal, 1:10,000; ref. 29) and mouse anti-TH (1:8000; 22941, Immunostar) antibodies were incubated on sections overnight at 4°C. Then, sections were incubated in biotinylated secondary antisera against either mouse (1:400, MilliporeSigma) or rabbit IgG (1:400, MilliporeSigma) followed by Vector ABC detection kit (Vector Laboratories). Antibody labeling was visualized by exposure to 0.5 mg/mL 3,3′ diaminobenzidine (DAB) and 0.03% H_2_O_2_ in Tris buffer. Sections were mounted on subbed slides, dehydrated to xylene, and coverslipped with Cytoseal (Richard-Allan Scientific).

### Immunofluorescence in rats.

For DAPI staining, an additional 3-minute incubation in Tris buffer with DAPI (1:500; Invitrogen) was performed. Primary antibodies used include rabbit anti-TH (1:4000; AB152, MilliporeSigma), mouse anti-pSyn (81a, monoclonal, 1:15,000; ref. 29). Secondary antibodies used included Alexa Fluor 568 goat anti–mouse IgG (1:500; Invitrogen) and Alexa Fluor 488 goat anti–rabbit IgG (1:500; Invitrogen).

### Medial terminal nucleus (MTN) counts of TH neurons in the SN in rats.

TH neurons from 3 sections of the SN, easily identified by proximity to the MTN of the accessory optic tract (−5.04 mm, −5.28 mm, and −5.52 mm relative to bregma), were quantified as previously described (88).

### Stereology in rats.

Microbrightfield stereological software (MBF Bioscience) was used to assess total population cell counts in the SN pars compacta (SNpc). The total number of stained neurons was calculated using optical fractionator estimations, and the variability within animals was assessed via the Gundersen coefficient of error (<0.1) (89).

### High-performance liquid chromatography (HPLC) in rats.

A dorsolateral striatal tissue punch was taken from both hemispheres. Frozen punches were placed individually in vials supercooled on dry ice and stored at −80°C until analysis. Tissue was homogenized and analyzed as described previously (90, 91).

### Mice.

Adult male and female C57Bl/6 mice (8–10 weeks of age, 20–32 g; Taconic Biosciences) were utilized at Ionis Pharmaceuticals and University of Pittsburgh. Mouse studies using human WT SNCA-PAC (Licensed from Mayo Foundation for Medical Education and Research) mice were maintained by Taconic and studies conducted at Ionis pharmaceuticals.

### Intrastriatal PFF injections in mice.

Mouse PFF studies were performed as described previously, including pSer129^+^ aggregate counts, dopaminergic cell counts, and wire hang task in mice (82). Purification of recombinant in vitro fibril assembly was performed as previously described (29, 82, 87). Mice were injected in the striatum with 5 μg of aSyn PFFs in 2 μL of dosing solution.

### I.c.v. ASO injection in mice.

Mice were injected into the right cerebral ventricle (i.c.v.) using a stereotactic device as previously described (82).

### Tissue processing in mice.

Following in-life completion, mice were euthanized by CO_2_ asphyxiation. Sections (2 mm) of different brain regions were collected for mRNA analysis. Brains were also cut sagittally in a 2 mm–thick slab for IHC.

### aSyn IHC in mouse brain tissue.

Sagittal brain sections were fixed in formalin for 24 hours, followed by sectioning as described in (82). Primary mouse anti-pSyn antibody SNL1 at 1:1000 as described in refs. 48 and 92.

### Mouse cortical cultures.

Mouse hippocampal neurons were cultured in 96-well plates (60,000 cells/cm^2^) and treated with either aSyn PFFs or PBS control at 7 DIV. ASOs were added 30 minutes following aSyn exposure at a final concentration of 2 μM. Ten days after initial treatment, neurons were lysed in Triton/DOC buffer (0.5% TX-100, 0.5% deoxycholic acid, 10 mM TRIS, and 100 mM NaCl; pH 8.0) containing phosphatase and protease inhibitors and were centrifuged for 5 minutes at 4°C at 1000*g*.

### aSyn Western blots in mouse hippocampal cultures.

The supernatant fractions were collected and total protein was assayed using the BCA method. Equal amounts of protein were separated on 4%–15% gels (Mini-PROTEAN, Bio-Rad) and transferred to nitrocellulose (0.22 μm). Blots were probed with Syn9027 (pan-aSyn; 1:10,000) in blocking buffer (TBS containing in 5% nonfat milk). As a loading control, blots were also probed with Beta III Tubulin (TUJ1; 1:1000; BioLegend). After incubation with infrared-labeled secondary antibody to mouse IgG (1:20,000; Li-Cor), blots were scanned using a Li-Cor Odyssey scanner.

### H2aX methods in mouse hippocampal cultures.

These methods were performed as in ref. 42 with H2aX detected with phospho-Histone H2A.X (Ser139; 20E3; Cell Signaling Technologies).

### Nonhuman primates.

The NHP (cynomolgus) study was performed at Covance laboratories GmbH. Covance Laboratories GmbH test facility is fully accredited by the Association for Assessment and Accreditation of Laboratory Animal Care (AAALAC). All procedures in this study plan are in compliance with the German Animal Welfare Act and are approved by the local IACUC, and performed as described previously (93). NHPs (*n* = 4) were administered, by i.t. bolus injection, 5 doses of ASO or aCSF on days 1, 14, 28, 56, and 84 with 1.0 mL dosing volume using a 35 mg/mL dosing solution, and they were euthanized on day 91.

### aSyn ELISA.

Roughly 50 mg of NHP brain tissue was added to 1 mL of RIPA buffer (Boston Bioproducts) with protease and phosphatase inhibitor tablets (Sigma-Aldrich) in a 2 mL Lysing Matrix D tube (MP Biomedicals). The samples were homogenized using an MP Fastprep-24 (MP Biomedicals), and total protein was quantified using the Pierce BCA Protein Assay kit (Thermo Fisher Scientific) and normalized to 1 mg/mL. Tissue samples were diluted either 1:100 (spinal cord), 1:2000 (brain tissue) or 1:10 (CSF) prior to aSyn protein determination. aSyn protein concentrations were measured using the LEGENDMAX Human Alpha Synuclein ELISA Kit (BioLegend) following the manufacturer’s protocol. CSF hemoglobin levels were also analyzed using the human hemoglobin ELISA kit (Bethyl Laboratories). This was done to assess the impact of blood contamination on the aSyn levels detected in CSF due to the high levels of aSyn contained in RBCs. Samples with greater than 1000 ng/mL HgB were excluded from the CSF analysis.

### ASO IHC in NHP tissue.

Immunohistochemical staining was performed on a Ventana *DISCOVERY ULTRA* autostainer (Ventana). Staining was done according to the manufacturer’s instructions. Briefly, 5 μm–thick sections were deparaffinized and rehydrated and were then subjected to antigen retrieval using Proteinase K (Dako) for 4 minutes, followed by incubation with a rabbit polyclonal anti-ASO antibody at 1: 10,000 dilution (Ionis, ASO 6651) for 60 minutes. The signal was detected with polymer-based secondary antibody Discovery OmniMap anti–rabbit HRP and Discovery ChromoMap DAB kits (Ventana, 760-4311). Control samples were also stained with rabbit IgG (Cell Signaling Technologies, 2792) as isotype controls. All slides were scanned on a 3DHISTECH Panoramic P250 Slide Scanner. ASO images were scanned at 20× magnification. All images were taken as screen shots from virtual slides. All images were analyzed with custom image analysis algorithms performed on the Visiopharm software platform. Target brain regions on each slide were manually outlined in Visiopharm to designate areas for algorithm analysis. In the Visiopharm analysis algorithm, ASO staining was assessed as positively stained region area broken down into 3 levels of intensity. A minimum threshold for ASO staining was determined by assessing background levels of DAB (brown) staining. A maximum threshold of 100% intensity of DAB staining was used. ASO staining ranges were determined independently per stain. This range was evenly divided into low-, medium-, and high-intensity bins; each area of staining was calculated as a percentage of the total outlined tissue area (brain region).

### SNCA-RNA in situ hybridization (ISH) staining in NHP tissue.

ISH staining was performed on a Leica Biosystems’ BOND RX Autostainer (Leica Biosystems) using mRNAscope 2.5 LS Reagent Kit—RED (Advanced Cell Diagnostics). Staining was done according to the manufacturer’s instructions. Briefly, 5 μm–thick sections were deparaffinized and rehydrated; they were then subjected to pretreatment, followed by specific probe-SNCA (ACD, 421318) hybridized to target mRNA. The signal was amplified using multiple steps, followed by hybridization to alkaline phosphatase–labeled (AP-labeled) probes and detection using Fast Red (ACD, 322150). The tissue quality was examined by positive control probe-PPIB (ACD, 424148), and background staining was assessed by negative control probe-dapB (ACD, 312038). All slides were scanned on a 3DHISTECH Panoramic P250 Slide Scanner. ISH images were scanned at 40× magnification. All images were taken as screen shots from virtual slides. All images were analyzed with custom image analysis algorithms performed on the Visiopharm software platform. Target brain regions on each slide were manually outlined in Visiopharm to designate areas for algorithm analysis. In the Visiopharm analysis algorithm, aSyn mRNA counts were determined by detecting and counting SNCA ISH-labeled dots within each region. These counts were normalized by the total outlined tissue area (brain region) in mm^2^.

### aSyn IHC in NHP tissue.

Immunohistochemical staining was performed on a Ventana *DISCOVERY ULTRA* autostainer (Ventana). Staining was done according to the manufacturer’s instructions. Briefly, 5 μm–thick sections were deparaffinized and rehydrated; they were then subjected to antigen retrieval using Ventana CC1 (EDTA pH 8) for 64 minutes, followed by incubation with a mouse monoclonal antibody aSyn211 (Santa Cruz Biotechnology Inc., sc-12767) at 0.25 μg/mL for 60 minutes. The signal was detected with polymer-based secondary antibody Discovery OmniMap anti–mouse HRP for 16 minutes. Control samples were also stained with mouse IgG (Cell Signaling Technologies) as isotype controls. All slides were scanned on a 3DHISTECH Panoramic P250 Slide Scanner. aSyn protein images were scanned at 20× magnification. All images were taken as screen shots from virtual slides. All images were analyzed with custom image analysis algorithms performed on the Visiopharm software platform. Target brain regions on each slide were manually outlined in Visiopharm to designate areas for algorithm analysis. In the Visiopharm analysis algorithm, aSyn protein staining was assessed as positively stained region area broken down into 3 levels of intensity. A minimum threshold for aSyn protein was determined by assessing background levels of DAB (brown) staining. A maximum threshold of 100% intensity of DAB staining was used. aSyn protein ranges were determined independently per stain. This range was evenly divided into low-, medium-, and high-intensity bins; each area of staining was calculated as a percentage of the total outlined tissue area (brain region).

### mRNA purification and analysis for mice, rats, andNHPs.

Approximately 10 mg of tissue per sample was homogenized in guanidinium isothiocynate. Total mRNA was purified further using a mini-mRNA purification kit (Qiagen). After quantitation, the tissues were subjected to real-time PCR analysis. The Life Technologies ABI StepOne Plus Sequence Detection System was employed. Briefly, 20 μL reverse transcription PCR (RT-PCR) reactions containing 5 μL of mRNA were run with the RNeasy 96 kit reagents (Qiagen), and the primer probe sets listed in the materials section were optimized from the manufacturer’s instructions. The following sequences of primers and probes were used: mouse *Snca*, 5′-GTCATTGCACCCAATCTCCTAAG-3′ (forward), 5′-GACTGGGCACATTGGAACTGA-3′ (reverse), and 5′-FAM-CGGCTGCTCTTCCATGGCGTACAAX- TAMRA-3′ (probe); rat *Snca*, 5′-GATGGGCAAGGGTGAAGAAG-3′ (forward), 5′-GCTAGGGTCCACAGGCATGT-3′ (reverse), and 5′-FAM-TACCCACAAGAGGGAAT-MGB-3′ (probe); human *SNCA*, 5′- TGGCAGAAGCAGCAGGAAA-3′ (forward), 5′-TCCTTGGTTTTGGAGCCTACA-3′ (reverse), and 5′-FAM-CAAAAGAGGGTGTTCTC-TAMRA-3′ (probe); mouse *cyclophilin A*, 5′-TCGCCGCTTGCTGCA-3′ (forward), 5′-ATCGGCCGTGATGTCGA-3′ (reverse), and 5′-FAM-CCATGGTCAACCCCACCGTGTTCX-TAMRA-3′ (probe); rat *cyclophilin A*, 5′- CCCACCGTGTTCTTCGACA-3′ (forward), 5′- AAACAGCTCGAAGCAGACGC-3′ (reverse), and 5′- CACGGCTGATGGCGAGCCCX-TAMRA-3′ (probe); NHP *cyclophilin A*, 5′- TGCTGGACCCAACACAAATG-3′ (forward), 5′- TGCCATCCAACCACTCAGTC-3′ (reverse), and 5′- TTCCCAGTTTTTCATCTGCACTGCCAX-TAMRA-3′ (probe). Target *SNCA* mRNA was then normalized to Cyclophilin A mRNA levels from the same mRNA sample. SNCA mRNA from ASO-treated animals was further normalized to the group mean of PBS-treated animals and expressed as percent control. All qPCR reactions were run in triplicate. Data were analyzed using Microsoft Excel (v14.4).

### SH-SY5Y cell culture assay.

SH-SY5Y cells (CRL-2266, ATCC) were cultured in growth medium at 37°C and 10% CO_2_. ASO was electroporated into cells by pulsing once at 160V for 6 mS with the ECM 830 instrument (Harvard Apparatus). After 24 hours, the cells were washed 1× with PBS before lysing for mRNA isolation and analysis. For each treatment condition, quadruplicate wells were tested.

### RNA purification and analysis for cell culture.

The mRNA was purified with a glass fiber filter plate (Pall, 5072) and chaotropic salts. The human *SNCA* message level was quantitated with qPCR on the QS7 instrument (Applied Biosystems). Total mRNA levels were measured with the Quant-iT RiboGreen mRNA reagent and used to normalize the *SNCA* data. Data were analyzed using Microsoft Excel (v14.4) and GraphPad Prism (v6).

### Data and materials availability.

No large-scale data sets were generated in this study. All ASO sequences and chemistries, as well as references to the synthesis, are included in the methods to allow for generation of these compounds. SNCA-PAC mice were licensed from Mayo Foundation for Medical Education and Research.

### Statistics.

Statistical tests were completed using either GraphPad Prism software (version 6, GraphPad) or Microsoft excel (v14.4). Data are expressed as mean ± SEM, unless otherwise noted. For 2-group comparisons, 2-tailed *t* tests were used. For comparisons involving more than 2 groups, 1-way ANOVA was used. Comparisons of multiple groups made across time points were analyzed using a 2-way ANOVA. When appropriate, post-hoc comparisons were made between groups using Tukey or Bonferonni post hoc tests. A ROUT analysis was performed to determine outliers using *Q* = 1%. The level of significance was set at *P* ≤ 0.05.

### Study approval.

All experimental procedures involving animals were approved by the Institutional Care and use Committee at Ionis Pharmaceuticals, Michigan State University, and University of Pennsylvania. All procedures in this study plan are in compliance with the German Animal Welfare Act and are approved by the local IACUC.

## Author contributions

The study was conceived by TAC and HBK. Experiments were designed by TAC, HBK, HZ, and KLP. ASOs were designed by TAC, HBK, and EES. Experiments were performed and analyzed by KLP, SC, TJC, HZ, AB, AM, CES, ES, EL, ACS, MW, MB, LJ, and JL. Animal work was carried out by KLP, HZ, TJC, BFD, IS, CES, KSC, and AB. ELISA/IHC was carried out by KLP, MW, MB, TJC, HZ, DQ, and BFD. HPLC was performed by JL. Fibrils were obtained from AW, and KCL, ES, CFB, KLP, TJC, DG, AW, and KCL gave conceptual advice. TAC and HBK wrote the manuscript.

## Supplementary Material

Supplemental data

## Figures and Tables

**Table 1:**
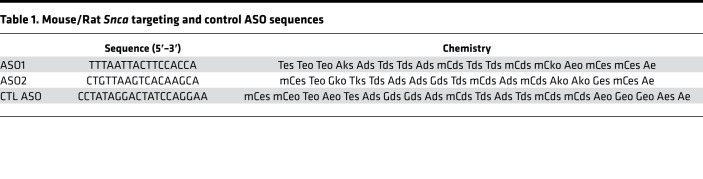
Mouse/Rat *Snca* targeting and control ASO sequences

**Table 2:**
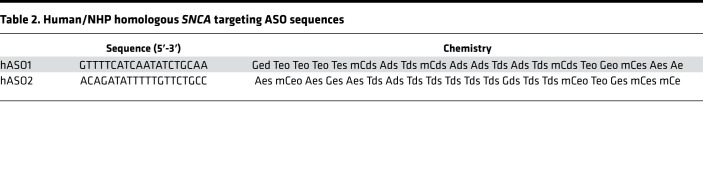
Human/NHP homologous *SNCA* targeting ASO sequences

## Version 1 03/08/2021

Electronic publication
